# Phase I dose escalation study of BI 836826 (CD37 antibody) in patients with relapsed or refractory B-cell non-Hodgkin lymphoma

**DOI:** 10.1007/s10637-020-00916-3

**Published:** 2020-03-14

**Authors:** Frank Kroschinsky, Jan Moritz Middeke, Martin Janz, Georg Lenz, Mathias Witzens-Harig, Reda Bouabdallah, Paul La Rosée, Andreas Viardot, Gilles Salles, Seok Jin Kim, Tae Min Kim, Oliver Ottmann, Joerg Chromik, Anne-Marie Quinson, Ute von Wangenheim, Ute Burkard, Andreas Berk, Norbert Schmitz

**Affiliations:** 1 Medical Department I, University Hospital at the Technical University of Dresden, Fetscherstr. 74, 01307 Dresden, Germany; 2 Experimental and Clinical Research Center, Max Delbrück Center for Molecular Medicine and Charité – Universitätsmedizin Berlin, Robert-Rössle-Straße 10, 13125 Berlin, Germany; 3 Department of Hematology and Oncology, University Hospital Muenster, Albert-Schweitzer-Campus 1, 48149 Münster, Germany; 4 Internal Medicine V: Hematology, Oncology and Rheumatology, University Hospital Heidelberg, Im Neuenheimer Feld 672, 69120 Heidelberg, Germany; 5 Department of Hematology, Institute Paoli Calmettes, 232 Boulevard de Sainte-Marguerite, 13009 Marseille, France; 6 Klinik für Innere Medizin II, Universitätsklinikum, Jena, Germany; 7 Present Address: Klinik für Innere Medizin II, Schwarzwald-Baar-Klinikum, Villingen-Schweningen, Germany; 8 Department of Internal Medicine III, University Hospital of Ulm, Albert-Einstein-Allee 23, 89081 Ulm, Germany; 9 Department of Hematology, University Hospital of South Lyon, 165 Chemin du Grand Revoyet, 69310 Pierre-Bénite, France; 10 Division of Haematology-Oncology, Department of Medicine, Samsung Medical Center, Sungkyunkwan University School of Medicine, 81 Irwon-ro, Irwon-dong, Gangnam-gu, Seoul, South Korea; 11 Department of Internal Medicine, Seoul National University Hospital, 101 Daehak-Ro Jongno-Gu, Seoul, 03080 South Korea; 12 Cancer Research Institute, Seoul National University College of Medicine, 101 Daehak-ro, Jongno-gu, Seoul, South Korea; 13 Division of Cancer and Genetics, Department of Haematology, Cardiff University, Heath Park, Cardiff, CF14 4XN UK; 14 Universitätsklinikum Frankfurt, Johann-Wolfgang-Goethe-Universität, Theodor-W.-Adorno-Platz 1, 60323 Frankfurt, Germany; 15 Boehringer Ingelheim Pharmaceuticals Inc., 900 Ridgebury Road, Ridgefield, CT 06877 USA; 16 Boehringer Ingelheim Pharma GmbH & Co. KG, Birkendorfer Str. 65, 88397 Biberach an der Riß, Germany; 17 ClinTriCare GmbH & Co. KG, Untere Illereicher Str. 10, 89281 Altenstadt, Germany

**Keywords:** BI 836826, CD37, Diffuse large B cell lymphoma, Non-Hodgkin lymphoma, Phase I, Relapsed

## Abstract

BI 836826 is a chimeric immunoglobulin G1 antibody targeting CD37, a tetraspanin transmembrane protein predominantly expressed on normal and malignant B cells. This phase I, open-label study used a modified 3 + 3 design to evaluate the safety, maximum tolerated dose (MTD), pharmacokinetics, and preliminary activity of BI 836826 in patients with relapsed/refractory B cell non-Hodgkin lymphoma (NHL; NCT01403948). Eligible patients received up to three courses comprising an intravenous infusion (starting dose: 1 mg) once weekly for 4 weeks followed by an observation period of 27 (Course 1, 2) or 55 days (Course 3). Patients had to demonstrate clinical benefit before commencing treatment beyond course 2. Forty-eight patients were treated. In the dose escalation phase (1–200 mg) involving 37 Caucasian patients, the MTD was 100 mg. Dose-limiting toxicities occurred in four patients during the MTD evaluation period, and included stomatitis, febrile neutropenia, hypocalcemia, hypokalemia, and hypophosphatemia. The most common adverse events were neutropenia (57%), leukopenia (57%), and thrombocytopenia (41%), and were commonly of grade 3 or 4. Overall, 18 (38%) patients experienced infusion-related reactions, which were mostly grade 1 or 2. Preliminary evidence of anti-tumor activity was seen; three patients responded to treatment, including one complete remission in a Korean patient with diffuse large B cell lymphoma. BI 836826 plasma exposure increased more than proportionally with increasing doses. BI 836826 demonstrated preliminary activity; the most frequent adverse events were hematotoxicity and infusion-related reactions which were manageable after amending the infusion schedule. Although BI 856826 will not undergo further clinical development, these results confirm CD37 as a valid therapeutic target in B cell NHL.

**Electronic supplementary material:**

The online version of this article (10.1007/s10637-020-00916-3) contains supplementary material, which is available to authorized users.

## Introduction

Non-Hodgkin lymphomas (NHLs) are a heterogeneous group of malignant disorders with variable clinical and biologic features that together caused over 248,000 deaths worldwide in 2018 [1, 2]. Most NHLs are of B cell origin, and the most common form, diffuse large B cell lymphoma (DLBCL), is an aggressive subtype that is readily curable with immunochemotherapy in the majority of patients but progresses rapidly if left untreated [1, 3]. In contrast, indolent follicular lymphoma (FL) is characterized by favorable prognosis but is still considered to be incurable in the vast majority of cases [4]. Although the addition of the anti-CD20 monoclonal antibody, rituximab, to the NHL therapeutic armamentarium more than 20 years ago greatly improved outcomes, prognosis remains poor for DLBCL patients who are not cured by first-line therapy or FL patients who suffer early progression [3, 4]. New treatments able to overcome resistance to standard immunochemotherapy are therefore urgently needed.

The tetraspanin CD37 is a transmembrane protein that is expressed on B-cells at multiple stages of development, from pre-B to peripheral mature B-cells [5, 6]. CD37 appears to play multiple roles in immune cells, including regulation of apoptosis/survival signaling, B/T cell interaction, and T cell proliferation [5, 7–9]. Notably, CD37 is highly expressed on malignant B cells, including most subtypes of NHL [10, 11], making it an attractive therapeutic target. Clinical studies with the anti-CD37 agents otlertuzumab (TRU-016) [12], IMGN529 [13], and AGS67E [14] suggest that targeting CD37 is a viable therapeutic strategy, with evidence of anti-tumor activity seen in patients with DLBCL, FL and other B cell NHLs.

BI 836826 is a chimeric immunoglobulin G1 (IgG1) monoclonal antibody that targets human CD37. It comprises a high-affinity mouse antibody to CD37 with an engineered CH2 domain to improve binding to human Fcγ receptors [6]. Preclinical studies demonstrated strong pharmacodynamic and antitumor effects of BI 836826 [6]. It showed high intrinsic proapoptotic activity accompanied by homotypic aggregation against malignant B cells and promoted antibody-dependent cell-mediated cytotoxicity (ADCC) against lymphoma cells. In blood samples from healthy volunteers, BI 836826 depleted normal B cells and spiked B-lymphoma cells more potently than rituximab. In experiments in vivo, BI836826 dose-dependently reduced peripheral B cells in CD37 transgenic mice and cynomolgous monkeys. It also suppressed tumor growth in a Ramos mouse B cell lymphoma model [6]. In a first-in-human study, BI 836826 showed evidence of anti-tumor activity in patients with relapsed or refractory chronic lymphocytic leukaemia (CLL) [15]. These findings warranted the evaluation of BI 836826 in patients with B cell NHL. Here we report the results of a phase I, dose-escalation study to evaluate the maximum tolerated dose (MTD), safety, and efficacy of BI 836826 in patients with relapsed or refractory B cell NHL (ClinicalTrials.gov identifier: NCT01403948).

## Patients and methods

### Patients

The study enrolled patients with relapsed or refractory NHL of B cell origin (mature B cell lymphoma according to the World Health Organization [16]) who were not considered candidates for intensive anti-lymphoma therapy. Patients were required to have either aggressive NHL and at least one (at least two for patients enrolled in France) prior anti-CD20-containing immunochemotherapeutic regimens, or indolent NHL with previous anti-CD20 therapy and at least two prior therapies. Other key requirements were age ≥ 18 years, Eastern Cooperative Oncology Group performance status (ECOG PS) of <2, and a life expectancy of ≥3 months. Patients in the expansion cohort were also required to have measurable disease on computed tomography (CT) scan.

Patients were ineligible if they had primary central nervous system (CNS) lymphoma or known CNS involvement, or a prior history of malignancy other than a mature B cell neoplasm, basal or squamous cell carcinoma of the skin, or carcinoma in situ of the uterine cervix or breast, unless free of disease and without treatment for at least 5 years. In addition, patients were excluded if they had inadequate organ function; a significant concurrent medical disease or condition; or chronic or ongoing infectious disease requiring treatment at enrollment or within the previous 2 weeks, including CMV viremia, HIV, or active hepatitis B or C.

The study was conducted in accordance with the Declaration of Helsinki and Good Clinical Practice guidelines, and the study protocol was approved by the Institutional Review Boards or Independent Ethics Committees of all participating institutions. Written informed consent was obtained from all patients.

### Study design and treatment

This was an open-label, single-arm, phase I dose-escalation study. The primary objective was to determine the MTD of BI 836826 in patients with NHL of B cell origin. Secondary objectives were to assess the safety and tolerability of BI 836826, to perform pharmacokinetic (PK) and pharmacodynamic (PD) analyses, and to evaluate efficacy.

BI 836826 was administered as an intravenous infusion, initially at a set rate of 83.4 mL/h. Following the occurrence of a Grade 4 infusion-related reaction (IRR), the protocol was amended, and the infusion rate was changed to a slowly increasing dosing schedule beginning at 10 mL/h and increasing every 30 min by 10 mL/h if tolerable, to a maximum of 80 mL/h.

Each patient could receive a maximum of three courses of treatment. The first treatment course was of 7 weeks duration and consisted of one infusion per week for 4 weeks, followed by a 27-day observation period (Supplemental Fig. 1). A second, similar course was administered if BI 836826 was well tolerated and no signs of disease progression were seen at the first response assessment during week 7. Patients received a third course, of up to 12 weeks duration, if they continued to tolerate BI 836826 well, and achieved a response (complete remission [CR], CR unconfirmed [CRu], partial remission [PR]) or stable disease according to CT assessment during the second treatment cycle. Premedication with acetaminophen/paracetamol, antihistamine, and glucocorticoid was mandatory at the start of therapy. Supportive care, such as granulocyte colony-stimulating factors and prophylactic treatment with antibiotics and antivirals was permitted according to local guidelines.

In the first part of the study, BI 836826 was administered to Caucasian patients in escalating dose tiers, starting at 1 mg. Single-patient cohorts were treated until a drug-related adverse event (AE) of Grade ≥ 2 occurred during the MTD evaluation period (the time from the first administration of BI 836826 until 7 days after the second administration). Subsequent cohorts were expanded to three patients following a fixed dose-escalation design with dose de-escalation steps. Dose escalation continued until the MTD was reached, defined as the highest dose of BI 836826 for which the incidence of dose-limiting toxicities (DLTs) was no more than 17% (i.e., one out of six patients) during the MTD evaluation period.

DLTs were defined as any drug-related Grade ≥ 3 non-hematologic AE, except IRRs. Complications resulting from hematologic AEs, such as bleeding due to thrombocytopenia or infection due to neutropenia, were classified as non-hematologic AEs and captured as DLTs. Although not deemed to be DLTs, hematologic AEs (e.g., neutropenia, thrombocytopenia and anemia) were considered for definition of the dose for further development.

Following the determination of the MTD in Caucasian patients, a separate cohort was to be enrolled into an expansion phase at the MTD. Initial enrollment into this cohort included Korean patients. Early results indicated that the tolerability of BI 836826 differed between Korean and Caucasian patients, as three of four Korean patients developed significant drug-related AEs. Consequently, the protocol was amended to explore lower dose levels in a designated escalation cohort of Korean patients, with the aim of defining an MTD in that population.

### Study assessments

The primary endpoint of the study was the determination of the MTD and the number of DLTs observed during the period from the first administration of BI 836826 until 7 days after the second administration. Other safety assessments included the incidence and severity of AEs, graded according to the Common Terminology Criteria for Adverse Events, Version 4.0, laboratory parameters, and physical examinations.

Secondary efficacy endpoints were: tumor size reduction; best overall response, according to Standardized or Revised Response Criteria for Malignant Lymphoma [17]; progression-free survival (PFS); and failure-free survival (FFS), defined as the time from first administration of BI 836826 until disease progression, death, or start of next NHL therapy.

The PK parameters of BI 836826 were evaluated using non-compartmental analysis methods and included maximum measured plasma concentration (C_max_); terminal half-life (t_1/2_); area under the plasma concentration-time curve over the time interval of one treatment course (AUC_0-tz_); area under the plasma concentration-time curve over the time interval from zero extrapolated to infinity (AUC_0-∞_); total plasma clearance (CL); and volume of distribution after intravenous infusion at steady state (V_ss_). Blood samples for PK analysis were taken in cycle 1 before the start of infusion (pre-dose) and at 5, 7, 9, 24, and 72 h, and shortly before the start of the next infusion at 168 h. BI 836826 levels were determined using a validated enzyme-linked immunosorbent assay.

Exploratory PD evaluations were conducted into the potential prognostic biomarker β-2 microglobulin, and to investigate the potential predictive value of polymorphisms in the Fc fragment of the IgG receptor (*FCGR*) gene. β-2 microglobulin levels were determined using a routine blood sample obtained at screening, while *FCGR* genotyping was conducted using DNA extracted from a blood sample obtained at the first treatment visit in Cycle 1.

### Statistical methods

Statistical analyses were descriptive, and no formal statistical tests were performed for the dose groups. Exploratory analysis of time-to-event endpoints (PFS, FFS) was conducted using Kaplan–Meier methods.

## Results

### Patients and treatment exposure

A total of 59 patients were enrolled, and 48 treated with BI 836826 in 12 centers across Germany, France, and the Republic of Korea between 25 January 2012 and 28 February 2018.

Thirty-seven Caucasian patients were treated in the dose escalation phase. The median age was 69.0 years (range, 25–83), and 70% of patients were male (Table 1). The patients had been extensively pretreated, with most having received between three and six prior treatments, and more than half had FL. Eleven Korean patients were treated. The median age was 63.0 years (range, 27–79), 36% were male, and more than half had DLBCL (Table 1). All Korean patients had received prior systemic therapy, with most having received two or three prior treatments.

**Table 1:** Patient baseline characteristics

Characteristic	Caucasian patients *N* = 37	Korean patients *N* = 11
Male, *n* (%)	26 (70.3)	4 (36.4)
Median age, years (range)	69.0 (25–83)	63.0 (27–79)
Race, *n* (%)		
White	33 (89.2)	0
Asian	0	11 (100)
Missing^a^	4 (10.8)	0
ECOG PS at baseline, *n* (%)		
0	9 (24.3)	4 (36.4)
1	21 (56.8)	7 (63.6)
2	7 (18.9)	0
Ann Arbor stage at screening, *n* (%)		
I	3 (8.1)	0
II	5 (13.5)	0
III	9 (24.3)	2 (18.2)
IV	19 (51.4)	9 (81.8)
Missing	1 (2.7)	0
Lymphoma subtype at screening, *n* (%)		
Follicular lymphoma	19 (51.4)	2 (18.2)
Diffuse large B-cell lymphoma	14 (37.8)	6 (54.5)
Mantle cell lymphoma	3 (8.1)	2 (18.2)
Other	1 (2.7)	1 (9.1)
Patients with prior stem cell transplant, *n* (%)	7 (18.9)	2 (18.2)
Patients refractory to last therapy, *n* (%)	23 (62.2)	5 (45.5)
Mean time since first diagnosis, years (SD)	5.6 (4.9)	2.5 (1.6)

*ECOG PS* Eastern Co-operative Oncology Group performance status, *SD* standard deviation

^a^Race data were not recorded for patients treated at study sites in France as per local law

All patients discontinued treatment; reasons for discontinuation among Caucasian patients were progressive disease (59.5%), AEs other than DLTs (13.5%), patient refusal to continue with trial medication (5.4%), completion of all 12 infusions (10.8%), or other reasons (8.1%); one patient (2.7%) was lost to follow-up. Progressive disease was the primary reason for discontinuation among the Korean patients (90.9%); the remaining patient discontinued after receiving the maximum number of infusions. The median number of infusions was 4 (range, 1–12) and 3 (range, 2–12) in Caucasian and Korean patients respectively; the mean cumulative BI 836826 dose was 365.0 mg (standard deviation [SD] = 343.9 mg) and 286.4 mg (SD = 201.2 mg), respectively.

### MTD and DLTs

For the Caucasian patients, dose escalation proceeded through 1 mg (*n* = 1), 3 mg (*n* = 4), 9 mg (*n* = 3), 25 mg (*n* = 4), 50 mg (*n* = 6), and 100 mg (*n* = 3) with no DLTs observed. One of three patients initially enrolled in the 200 mg cohort experienced DLTs (Grade 3 oral herpes, stomatitis, and febrile neutropenia). The 200 mg cohort was subsequently expanded to a total of seven patients (one of six patients in the initial cohort withdrew consent and was replaced), and although no further DLTs were reported, five of the seven patients experienced Grade 3/4 leukopenia and/or neutropenia lasting more than 1 week and requiring the next infusion to be delayed (Table 2). As a result, the 200 mg dose was considered to have exceeded the MTD. Dose de-escalation was subsequently undertaken.

**Table 2:** DLTs and hematologic abnormalities in Caucasian patients during the MTD evaluation period

Dose level (mg)	Treated patients	Evaluable patients	DLTs (*n*)	Severe hematologic abnormalities based on laboratory data (*n*)
200	7^a^	6	Grade 3 oral herpes, stomatitis, and febrile neutropenia (1)	Grade 4 neutropenia, lymphopenia, thrombocytopenia, and/or leukopenia (5)
150	6	6	Grade 4 hypophosphatemia (2), Grade 3 hypokalemia and hypocalcemia (1)	Grade 4 neutropenia and/or leukopenia (4)
100^b^	6^c^	6	None	None
3	4^d^	3	None	Grade 4 leukopenia and neutropenia

*DLTs* dose-limiting toxicities, *MTD* maximum tolerated dose

^a^One patient withdrew consent during the MTD evaluation period and was replaced

^b^Determined to be the MTD

^c^Three patients in initial cohort plus three additional patients to determine the MTD

^d^One patient was replaced because they were not evaluable for DLTs in the MTD evaluation period

Three of six patients treated at a dose of 150 mg experienced a DLT during the MTD evaluation period (grade 4 hypophosphatemia [*n* = 2] and grade 3 hypokalemia and hypocalcemia [*n* = 1]). These AEs were resolved within 24 h with supportive care. In addition, four of the six patients experienced grade 3/4 leukopenia and/or neutropenia lasting >7 days after the MTD evaluation period, suggesting that this dose exceeded the MTD. The next lower dose level (100 mg) was subsequently expanded with an additional three patients (to a total of six patients). None of the six patients treated at this dose experienced a DLT during the MTD evaluation period, and the 100 mg dose was therefore defined as the MTD.

Three of four patients enrolled into the 100 mg expansion cohort in Korean patients developed significant drug-related AEs (infections associated with grade 4 neutropenia). Following the protocol amendment, a separate MTD was to be defined for Korean patients based on an escalation scheme starting with 50 mg of BI 836826 administered weekly for 4 weeks, with 75 mg as the next dose step. Of seven patients treated with 50 mg BI 836826 (one patient had violated the exclusion criteria and was replaced), one experienced a DLT of grade 3 herpes zoster virus, and the MTD was not reached. However, prior to the enrollment of any further patients, the clinical development of BI 836826 was terminated, and the study was closed.

### Safety

All patients experienced at least one AE. Among Caucasian patients, 7 (18.9%), 21 (56.8%) and 8 (21.6%) experienced a highest AE grade of 3, 4 and 5, respectively. None of the fatal AEs were considered related to BI 836826 (lymphoma progression [*n* = 6]; pulmonary edema [*n* = 2]). Of the Korean patients, 1 (9.1%), 8 (72.7%) and 2 (18.2%) had a highest AE grade of 3, 4 and 5, respectively. Neither of the fatal AEs (pneumonia and malignant pleural effusion) were considered related to study drug by the investigator.

**Fig. 1:**
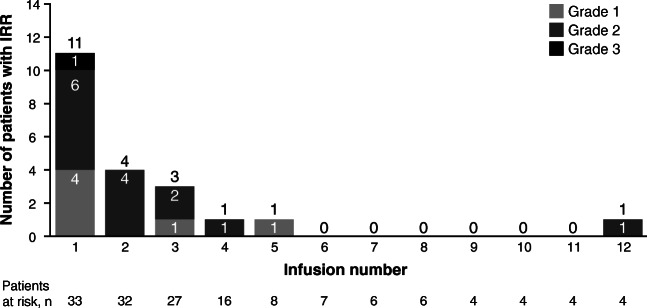
Incidence and severity of infusion-related reactions over time

The frequency of treatment-related AEs was 94.6% and 100% among the Caucasian and Korean patients, respectively (Table 3). The most frequent treatment-related grade 3/4 AEs among Caucasian patients were hematotoxicities and IRRs (8.1%; Table 3). Most IRRs were grade 1/2; one patient had a grade 4 IRR, which led to a protocol amendment resulting in a change in the infusion schedule. If necessary, the initial dose could also be split into two infusions. The most common symptoms associated with IRRs were chills and pyrexia. Following the change in infusion schedule, two patients had grade 3 IRRs. The incidence of IRRs was highest in cycle 1, and decreased over time (Fig. 1).

**Table 3:** Treatment-related adverse events occurring in >10% of patients

	Caucasian patients *n* = 37		Korean patients *n* = 11	
	Any grade *n* (%)	Grade 3/4 *n* (%)	Any grade *n* (%)	Grade 3/4 *n* (%)
Any treatment-related AE	35 (94.6)	29 (78.4)	11 (100.0)	11 (100.0)
Leukopenia	21 (56.8)	20 (54.1)	–	–
Neutropenia^a^	21 (56.8)	20 (54.1)	8 (72.7)	8 (72.7)
Thrombocytopenia	15 (40.5)	5 (13.5)	5 (45.5)	1 (9.1)
Febrile neutropenia	2 (5.4)	2 (5.4)	6 (54.5)	6 (54.5)
Infusion-related reaction	15 (40.5)	3 (8.1)	3 (27.3)	0
Chills	13 (35.1)	0	3 (27.3)	0
Lymphopenia	10 (27.0)	10 (27.0)	–	–
Decreased CD4 lymphocytes	8 (21.6)	8 (21.6)	–	–
Anemia	6 (16.2)	2 (5.4)	1 (9.1)	1 (9.1)
Fatigue	6 (16.2)	0	3 (27.3)	0
Pyrexia	4 (10.8)	0	–	–
Elevated C-reactive protein	4 (10.8)	0	–	–
Hypophosphatemia	4 (10.8)	2 (5.4)	–	–
Tachycardia	4 (10.8)	0	–	–
Pneumonia	–	–	2 (18.2)	2 (18.2)
Headache	1 (2.7)	0	2 (18.2)	0
Decreased neutrophil count^a^	2 (5.4)	2 (5.4)	3 (27.3)	3 (27.3)

*AE* adverse event, −, not listed, ^a^Different preferred terms, no difference per definition

Similar treatment-related Grade 3/4 AEs were reported in Korean patients, although the incidence of certain AEs differed considerably (Table 3). In particular, treatment-related grade 3/4 febrile neutropenia was much more common in Korean patients than in Caucasian patients (54.5% vs 5.4%).

Four Caucasian patients (10.8%) had serious AEs (SAEs) that were considered treatment-related, including bronchospasm, respiratory failure, IRR, sepsis, atrial fibrillation, oral herpes, febrile neutropenia, and stomatitis (none occurred in more than one patient). Six Korean patients (54.5%) experienced drug-related SAEs. These included five cases of febrile neutropenia (one patient experienced two separate episodes), two of pneumonia, and one case each of neutropenia, herpes zoster, septic shock, and bacteremia.

Eight Caucasian patients (21.6%) had AEs that resulted in discontinuation of study drug. The only AE leading to treatment discontinuation in more than one patient was IRR (two patients [5.4%]; both of these patients were treated prior to the change in the infusion schedule). No AEs occurred in Korean patients that led to treatment discontinuation. Two Caucasian patients and one Korean patient received dose reductions due to AEs.

Based on laboratory data, 20 Caucasian patients (54.1%) experienced grade 4 neutropenia lasting 1 week or longer (a total of 48 neutropenia episodes lasting ≥1 week; median 2.0 neutropenia episodes/patient). Eight patients (21.6%) had grade 4 neutropenia and a concomitant infection, of which four (10.8%) were grade 3/4. Prolonged grade 3/4 neutropenia of 1 week or longer was seen in all dose cohorts except 1 mg. At the MTD (100 mg), one of six patients had one episode of grade 4 neutropenia that lasted 11 days and delayed the administration of the next BI 836826 infusion. This episode did not require a dose reduction or discontinuation of the study drug. The platelet and white blood cell counts for a representative patient are shown in Supplemental Fig. 2. Among the Korean patients, 10 (90.9%) had grade 4 neutropenia episodes lasting more than 1 week (a total of 23 neutropenia episodes lasting ≥1 week, with a median of 2 neutropenia episodes per patient). Four patients (36.4%) had grade 4 neutropenia and a concomitant infection, all of which were grade 3/4.

Eight Caucasian patients, all receiving BI 836826 doses of 100 mg or higher, experienced 11 grade ≥ 3 infection episodes. Of these, two were considered drug-related (one case of sepsis and one episode of oral herpes), and dose reduction was required for the case of oral herpes. Among the Korean patients, six experienced grade ≥ 3 infections; three of seven patients in the 50 mg dose cohort, and three of four patients in the 100 mg dose cohort. All were considered to be DLTs. One patient died as a result of pneumonia.

### Efficacy

**Fig. 2:**
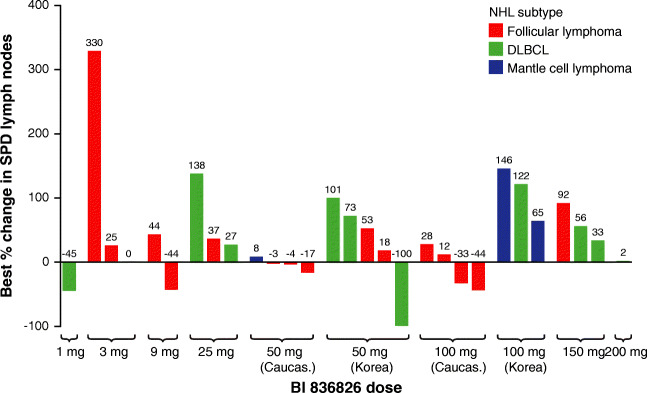
Best percentage change from baseline in the sum of product of diameters (SPD) of the indicator lesions, based on imaging data

In total, 21 Caucasian and eight Korean patients were evaluated for best change in the sum of the product of diameters (SPD) of their indicator lesions. The median best percentage change from baseline in SPD of indicator lesions was 11.8% (range, −44.7 to 329.8%) for Caucasian patients and 68.7% (range, −100.0 to 146.3%) for Korean patients (Fig. 2).Data from 32 Caucasian patients and 11 Korean patients were evaluable for best overall response (Table 4). According to investigator assessment based on imaging data, one Korean patient (50 mg dose group; DLBCL) achieved CR, while two Caucasian patients (100 mg dose group; FL and mantle cell lymphoma) achieved PR. Nine Caucasian patients (24.3%) and one Korean patient (9.1%) experienced stable disease. Taking into account all assessment methods (physical or imaging assessment by the investigator), a further two Caucasian patients (50 mg dose cohort and 100 mg dose cohort; both FL) had a PR. In the overall population, median PFS and FFS were 47 days (25th and 75th percentiles; 24 days, 169 days), and 46 days (24 days, 116 days), respectively.

**Table 4:** Best overall response in Caucasian and Korean patients

Patients with response, *n* (%)	Caucasian patients									Korean patients			Total
	BI 836826 dose									BI 836826 dose			
	1 mg	3 mg	9 mg	25 mg	50 mg	100 mg	150 mg	200 mg	Total	50 mg	100 mg	Total	
	*n* = 1	*n* = 4	*n* = 3	*n* = 4	*n* = 6	*n* = 6	*n* = 6	*n* = 7	*n* = 37	*n* = 7	*n* = 4	*n* = 11	*n* = 48
ORR	0	0	0	0	0	2 (33.3)	0	0	2 (5.4)	1 (14.3)	0	1 (9.1)	3 (6.3)
CR	0	0	0	0	0	0	0	0	0	1 (14.3)	0	1 (9.1)	1 (2.1)
CRu	0	0	0	0	0	0	0	0	0	0	0	0	0
PR	0	0	0	0	0	2 (33.3)	0	0	2 (5.4)	0	0	0	2 (4.2)
SD	1 (100)	1 (25.0)	1 (33.3)	0	3 (50.0)	1 (16.7)	0	2 (28.6)	9 (24.3)	1 (14.3)	0	1 (9.1)	10 (20.8)
PD	0	2 (50)	2 (66.7)	4 (100.0)	2 (33.3)	2 (33.3)	5 (83.3)	4 (57.1)	21 (56.8)	5 (71.4)	4 (100.0)	9 (81.8)	30 (62.5)
NE	0	1 (25.0)	0	0	1 (16.7)	1 (16.7)	1 (16.7)	1 (14.3)	5 (13.5)	0	0	0	5 (10.4)

*ORR* overall response rate, *CR* complete remission, *CRu* complete remission unconfirmed, *PR* partial remission, *SD* stable disease, *PD* progressive disease, *NE* not evaluable

### Pharmacokinetics

Non-compartmental PK parameters, analyzed for the combined population of Caucasian and Korean patients, were determined after the first intravenous infusion of BI 836826 for all patients in the study (Table 5). Maximum plasma concentration increased with dose up to 100 mg, but not in a dose-proportional manner. The highest individual value for C_max_ was reached in the 100 mg dose group (Supplemental Fig. 3). The volume of distribution (V_ss_) slightly decreased from 5.07 L in the 25 mg dose group to 2.82 L in the 100 mg dose cohort, and increased to 4.50 L for 200 mg, displaying overall a rather stable level and indicating that BI 836826 was primarily distributed in plasma at steady state.

**Table 5:** Overview of PK parameters after the first intravenous infusion of 1 mg to 200 mg BI 836826, for the combined population of Caucasian and Korean patients

PK parameter	BI 836826 dose							
	1 mg	3 mg	9 mg	25 mg	50 mg	100 mg	150 mg	200 mg
	*n* = 1	*n* = 4	*n* = 3	*n* = 4	*n* = 7	*n* = 10	*n* = 6	*n* = 7
C_max, norm_ (ng/mL/mg)	NC	88.6	150	176	206	302	208	210
AUC_0-tz_ (ng•h/mL)	NC	1670 (216)	13,100 (135)	75,000 (65.3)	204,000 (166)	770,000 (137)	1,520,000 (28.5)	1,870,000 (74.9)
t_1/2_ (hours)	NC	NC	NC	8.95 (112)	15.9 (62.2)	27.6 (108)	22.3 (41.8)	33.8 (122)
AUC_0-∞,norm_ (ng•h/mL/mg)	NC	NC	NC	2990	6990	15,200	10,200	12,600
CL (mL/min)	NC	NC	NC	5.58 (90.6)	2.38 (74.6)	1.10 (101)	1.63 (28.4)	1.32 (84.9)
V_ss_ (L)	NC	NC	NC	5.07 (13.1)	3.68 (33.9)	2.82 (26.6)	3.92 (20.8)	4.50 (22.8)

Data are shown as geometric mean (coefficient of variation, %)

*AUC*_*0-tz*_ area under the curve over the time interval of one treatment course, *AUC*_*0-∞*_ area under the curve over the time interval from zero extrapolated to infinity, *CL* clearance, *C*_*max*_ maximum concentration, *norm* normalized, *NC* not calculated, *PK* pharmacokinetic, *t*_*1/2*_ apparent terminal half-life of the analyte, *V*_*ss*_ volume of distribution at steady state

T_1/2_ was variable, ranging from a mean of 8.95 h in the 25 mg dose cohort to 33.8 h in the 200 mg dose group. A preliminary comparison of PK data from patients who received 100 mg BI 836826 suggested that Korean patients achieved approximately 4-fold higher plasma exposure to BI 836826 than Caucasian patients (Table 6).

**Table 6:** Comparison of PK parameters in Caucasian and Korean patients after the first intravenous infusion of 100 mg BI 836826

PK parameter	Caucasian patients	Korean patients
	*n* = 6	*n* = 4
C_max, norm_ (ng/mL/mg)	207	532
AUC_0-tz_ (ng•h/mL)	419,000 (106)	1,920,000 (24.7)
	*n* = 3	*n* = 3
AUC_0-∞_ (ng•h/mL/mg)	829,000 (63.9)	2,780,000 (61.5)
AUC_0-∞,norm_ (ng•h/mL/mg)	8290	27,800

Data are shown as geometric mean (coefficient of variation, %)

*AUC*_*0-tz*_ area under the curve over the time interval of one treatment course, *AUC*_*0-∞*_ area under the curve over the time interval from zero extrapolated to infinity, *C*_*max*_ maximum concentration, *norm* normalized, *PK* pharmacokinetic

### Pharmacodynamics

Two potential prognostic biomarkers were investigated in the study: β-2 microglobulin and *FCGR*. At screening, 43.8% of patients had elevated serum β-2 microglobulin levels (≥3.5 mg/L). No correlation was observed between baseline β-2 microglobulin levels and best percentage change in SPD of indicator lesions. Similarly, no association was observed between *FCGR* genotypes (polymorphisms FCGR2A, FCGR3A) and best overall response.

## Discussion

This study defined an MTD and demonstrated preliminary activity of BI 836826 in patients with B cell NHL, thus further validating CD37 as a therapeutic target in this setting. Notwithstanding the fact that few of the 48 treated patients received dose levels where activity may be expected, signals of efficacy were observed in this study. Out of 48 treated patients, one Korean patient had a best overall response of CR, and two Caucasian patients had a best overall response of PR, with SD the best overall response in 10 more patients.

The MTD in Caucasian patients was defined as 100 mg of BI 836826 administered weekly for 4 weeks. The clinical development of BI 836826 was stopped shortly after the 50 mg Korean cohort was completed, which prevented formal establishment of the MTD of BI 836826 in Korean patients. The MTD in this study was lower than observed in a previous phase I trial undertaken in patients with CLL [15]. While a formal MTD of BI 836826 in patients with CLL was not determined, doses of 400 mg administered every 2 weeks were tolerable and recommended for further development. The discrepancy between studies could be related to differences in target expression in different patient populations leading to different pharmacokinetic behavior due to target-mediated drug disposition. In both settings, the half-life of BI 836826 was short but clearance was more rapid in CLL than NHL. As reduction of circulating B cells and other blood cells expressing CD37 (e.g. T cells) underpins the mechanism of action of BI 826826 in both CLL and NHL, differences in clearance rate probably reflect the differences in the expression profile of CD37 in the two diseases, with higher expression in blood, spleen and bone marrow of patients with CLL compared to NHL. The short half-life of BI 826826 was unlikely to be attributable to its chimerism. While anti-drug antibodies were detectable in 19% of patients by the end of treatment, they were mostly detected after several infusions. However, rapid clearance was already observed after the first administration.

All Caucasian patients treated in the dose-escalation phase experienced at least one AE. These AEs were most frequently hematologic, particularly leukopenia, neutropenia, and thrombocytopenia, commonly of Grade 3/4. The occurrence of neutropenia and leukopenia tended to increase with increasing dose and was dose-limiting at the 200 mg and 150 mg doses. Although growth factor support was permitted, prolonged Grade 4 neutropenia lasting at least 1 week was seen in 54% of Caucasian patients and 91% of Korean patients, including eight cases of Grade 4 neutropenia and a Grade 3/4 concomitant infection. The tolerability profile of BI 836826 in this study was similar to that observed in patients with CLL [15].

The rapid kinetics of neutropenia and thrombocytopenia, with steep declines in neutrophil and platelet counts immediately after administration of BI 836826 and recovery prior to the next infusion 7 days later (Supplemental Fig. 2) suggest that cytopenia may be related to direct effects of BI 836826 on mature peripheral leukocytes and platelets, and not due to toxicity on bone marrow precursor cells. CD37 is known to be expressed in megakaryocytes, platelets, and neutrophils [18–20]; further, preclinical data suggest that BI 836826 does not impact on myeloid progenitor colony formation (unpublished data).

Fifteen Caucasian patients (40.5%) and three Korean patients (27.3%) experienced IRRs, with one Caucasian patient experiencing a Grade 4 event under the initial protocol-defined infusion rate of 83.4 mL/h over 3 h. These events occurred despite mandatory treatment with acetaminophen/paracetamol, antihistamine, and glucocorticoid prior to the infusion.

While this study suggests that BI 836826 may have a different tolerability profile between Caucasian and Korean patients, it must be noted that only 11 Koreans were treated. Hence the observations may merely reflect the small sample size. Nevertheless it was noteworthy that the PK of BI 836826 in Korean patients differed considerably from the PK seen in Caucasian patients. Exposure and maximum plasma concentration levels reached after intravenous administration of the 100 mg dose showed an almost 4-fold increase compared with the exposure in Caucasian patients. The finding of PK variability between Korean and Caucasian patients may be attributable to possible differences in drug metabolism between East Asians and Caucasians, which is a common phenomenon that may be related to differences in allelic frequencies of drug metabolism genes such as *CYP2D6* and the *CYP2C* subfamily [21].

Although the sponsor has decided not to pursue further clinical development of BI 836826, primarily due to changes in strategy reflecting recent changes in the treatment landscape for CLL, the results of this study support further clinical investigation of CD37 as a therapeutic target in B cell NHL. There was high rates of treatment-related AEs, particularly hematotoxicities, and this needs to be considered for future clinical development programs targeting CD37. However, recent promising results from a phase I/II study of ^177^Lu-lilotomab satetraxetan, an anti-CD37 antibody-radionuclide conjugate, in which 65% of patients with FL responded, including CR in 24% of patients, also confirm the value of targeting CD37 [22]. As with our study, the incidence of Grade 3/4 neutropenia (53%) was high; grade 3/4 thrombocytopenia was also common (48%). Further clinical investigation of AGS57E is ongoing [14], along with preclinical studies investigating CD37 as a target for chimeric antigen receptor T cell therapy [14, 23].

## Electronic supplementary material

ESM 1 (PDF 236 kb)

## Data Availability

The clinical study report (including appendices, but without line listings) and other clinical documents related to this study may be accessed on request. Prior to providing access, the documents and data will be examined, and, if necessary, redacted and de-identified to protect the personal data of study participants and personnel, and to respect the boundaries of the informed consent of the study participants. See https://trials.boehringer-ingelheim.com/data_sharing/sharing.html#accordion-1-2 for further details. Bona fide, qualified scientific and medical researchers may request access de-identified, analyzable patient-level study data, together with documentation describing the structure and content of the datasets. Researchers should use https://clinicalstudydatarequest.com/ to request access to raw data from this study.
