# Correction to: Phase I dose escalation study of BI 836826 (CD37 antibody) in patients with relapsed or refractory B-cell non-Hodgkin lymphoma

**DOI:** 10.1007/s10637-020-00949-8

**Published:** 2020-05-31

**Authors:** Frank Kroschinsky, Jan Moritz Middeke, Martin Janz, Georg Lenz, Mathias Witzens-Harig, Reda Bouabdallah, Paul La Rosée, Andreas Viardot, Gilles Salles, Seok Jin Kim, Tae Min Kim, Oliver Ottmann, Joerg Chromik, Anne-Marie Quinson, Ute von Wangenheim, Ute Burkard, Andreas Berk, Norbert Schmitz

**Affiliations:** 1 Medical Department I, University Hospital at the Technical University of Dresden, Fetscherstr. 74, 01307 Dresden, Germany; 2 Experimental and Clinical Research Center, Max Delbrück Center for Molecular Medicine and Charité – Universitätsmedizin Berlin, Robert-Rössle-Straße 10, 13125 Berlin, Germany; 3 Department of Hematology and Oncology, University Hospital Muenster, Albert-Schweitzer-Campus 1, 48149 Münster, Germany; 4 Internal Medicine V: Hematology, Oncology and Rheumatology, University Hospital Heidelberg, Im Neuenheimer Feld 672, 69120 Heidelberg, Germany; 5 Department of Hematology, Institute Paoli Calmettes, 232 Boulevard de Sainte-Marguerite, 13009 Marseille, France; 6 Klinik für Innere Medizin II, Universitätsklinikum, Jena, Germany; 7 Klinik für Innere Medizin II, Schwarzwald-Baar-Klinikum, Villingen-Schweningen, Germany; 8 Department of Internal Medicine III, University Hospital of Ulm, Albert-Einstein-Allee 23, 89081 Ulm, Germany; 9 Department of Hematology, University Hospital of South Lyon, 165 Chemin du Grand Revoyet, 69310 Pierre-Bénite, France; 10 Division of Haematology-Oncology, Department of Medicine, Samsung Medical Center, Sungkyunkwan University School of Medicine, 81 Irwon-ro, Irwon-dong, Gangnam-gu, Seoul, South Korea; 11 Department of Internal Medicine, Seoul National University Hospital, 101 Daehak-Ro Jongno-Gu, Seoul, 03080 South Korea; 12 Cancer Research Institute, Seoul National University College of Medicine, 101 Daehak-ro, Jongno-gu, Seoul, South Korea; 13 Division of Cancer and Genetics, Department of Haematology, Cardiff University, Heath Park, Cardiff, CF14 4XN UK; 14 Universitätsklinikum Frankfurt, Johann-Wolfgang-Goethe-Universität, Theodor-W.-Adorno-Platz 1, 60323 Frankfurt, Germany; 15 Boehringer Ingelheim Pharmaceuticals Inc., 900 Ridgebury Road, Ridgefield, CT 06877 USA; 16 Boehringer Ingelheim Pharma GmbH & Co. KG, Birkendorfer Str. 65, 88397 Biberach an der Riß, Germany; 17 ClinTriCare GmbH & Co. KG, Untere Illereicher Str. 10, 89281 Altenstadt, Germany

**Correction to: Invest New Drugs**

**https://doi.org/10.1007/s10637-020-00916-3**

The article Phase I dose escalation study of BI 836826 (CD37 antibody) in patients with relapsed or refractory B-cell non-Hodgkin lymphoma, written by Frank Kroschinsky, Jan Moritz Middeke, Martin Janz, Georg Lenz, Mathias Witzens-Harig, Reda Bouabdallah, Paul La Rosée, Andreas Viardot, Gilles Salles, Seok Jin Kim, Tae Min Ki, Oliver Ottmann, Joerg Chromik, Anne-Marie Quinson, Ute von Wangenheim, Ute Burkard, Andreas Berk, Norbert Schmitz, was originally published electronically on the publisher’s internet portal on 14 March 2020 without open access. With the author(s)’ decision to opt for Open Choice the copyright of the article changed on May 2020 to © The Author(s) 2020 and the article is forthwith distributed under a Creative Commons Attribution 4.0 International License (https://creativecommons.org/licenses/by/4.0/), which permits use, sharing, adaptation, distribution and reproduction in any medium or format, as long as you give appropriate credit to the original author(s) and the source, provide a link to the Creative Commons licence, and indicate if changes were made.

The original article has been corrected

