# Supplementary material for: Phase I dose escalation study of BI 836826 (CD37 antibody) in patients with relapsed or refractory B-cell non-Hodgkin lymphoma
Source: Invest New Drugs. 2020 Mar 14;38(5):1472–82. doi: 10.1007/s10637-020-00916-3 (PMC7497676; doi:10.1007/s10637-020-00916-3)
Supplement: Supplementary file 1 — (PDF 236 kb) [file 10637_2020_916_MOESM1_ESM.pdf]

SUPPLEMENTARY MATERIAL

Supplemental Fig. 1 Treatment schedule

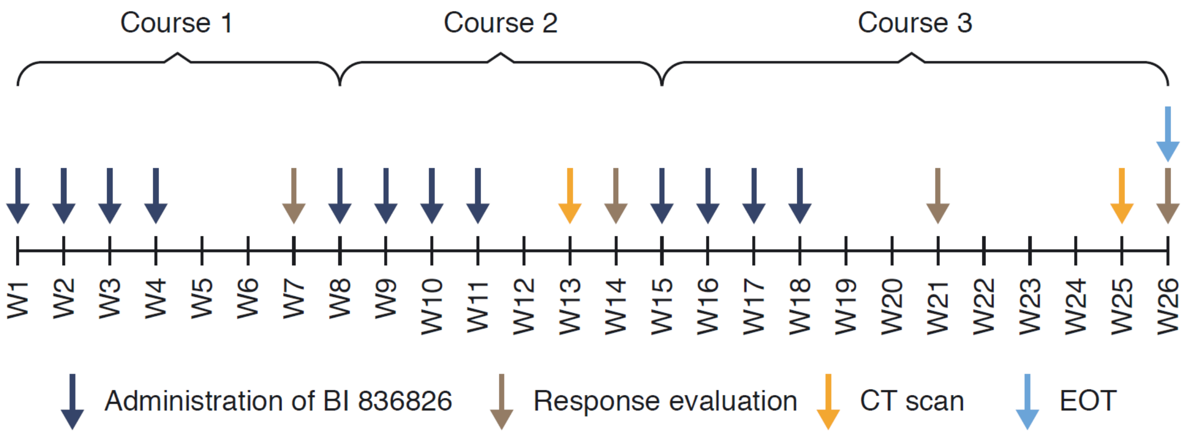

CT, computed tomography; *EOT*, end of treatment; *W*, week

Supplemental Fig. 2 Platelet and white blood cell counts for patient 505 treated with BI 836826 100 mg during Cycles 1 and 2

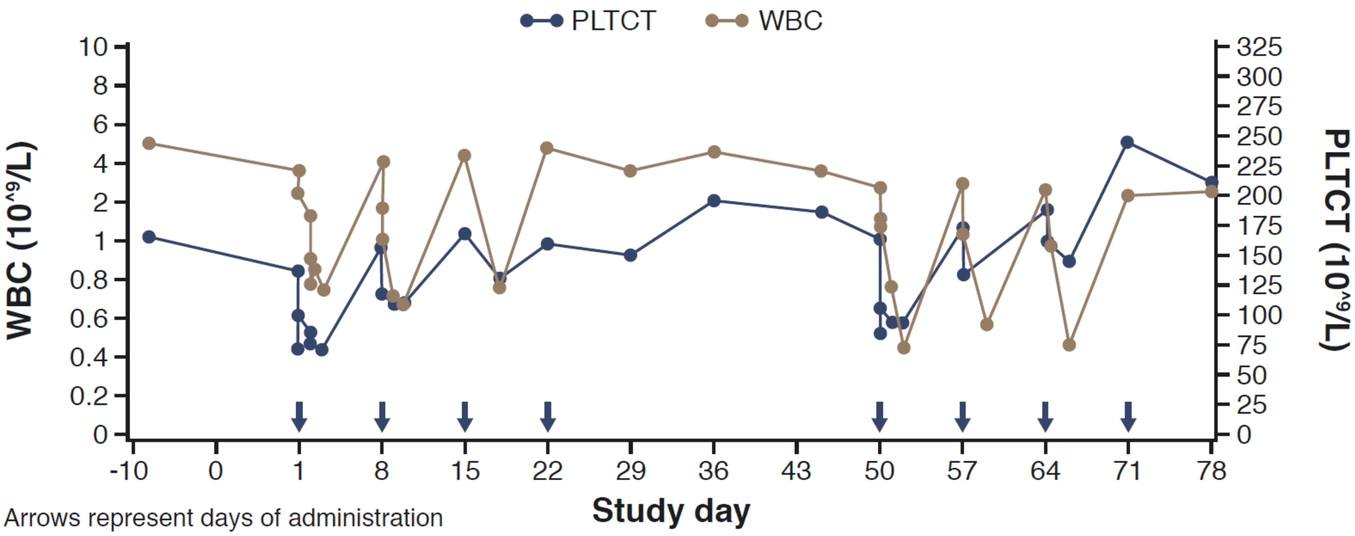

Arrows represent days of administration

PLTCT, platelet count; WBC, white blood cell count

Supplemental Fig. 3 Comparison of individual and geometric mean $C_{max, norm}$ values after the first infusion of BI 836826 over all dose groups from 3 mg to 200 mg (Caucasian and Korean patients combined)

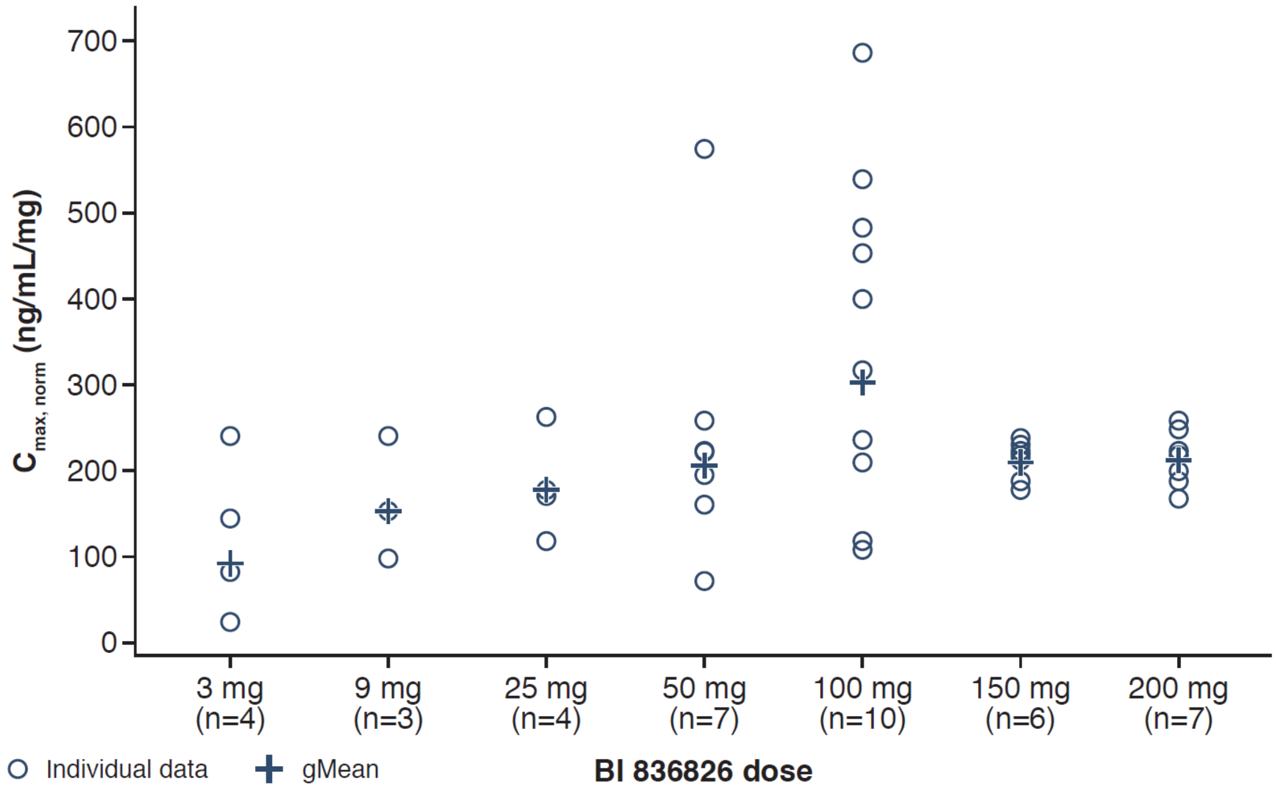

gMean, geometric mean
